# Dynamic changes in electrical brain activity during passive learning of foreign speech sound discrimination

**DOI:** 10.1093/cercor/bhaf181

**Published:** 2025-07-15

**Authors:** Qin Li, Jari L O Kurkela, Kaisa Lohvansuu, Jarmo A Hämäläinen, Xueqiao Li, Weiyong Xu, Piia Astikainen

**Affiliations:** Department of Psychology, University of Jyväskylä, PO Box 35, Mattilanniemi 6, Jyväskylä FI-40014, Finland; Department of Psychology, University of Jyväskylä, PO Box 35, Mattilanniemi 6, Jyväskylä FI-40014, Finland; Department of Teacher Education, University of Jyväskylä, PO Box 35, Alvar Aallon katu 9, Jyväskylä FI-40014, Finland; Department of Psychology, University of Jyväskylä, PO Box 35, Mattilanniemi 6, Jyväskylä FI-40014, Finland; Department of Psychology, University of Jyväskylä, PO Box 35, Mattilanniemi 6, Jyväskylä FI-40014, Finland; Department of Psychology, University of Jyväskylä, PO Box 35, Mattilanniemi 6, Jyväskylä FI-40014, Finland; Department of Psychology, University of Jyväskylä, PO Box 35, Mattilanniemi 6, Jyväskylä FI-40014, Finland

**Keywords:** change detection, foreign speech, multivariate pattern analysis, passive auditory exposure, phonetic learning

## Abstract

Previous brain research on phonetic learning of foreign speech sounds has focused on learning outcomes, mostly neglecting the dynamical neural changes during learning. In the present study, Finnish-speaking participants listened passively to a repeated presentation of vowel /a/ with infrequent changes in Mandarin tone for 2 h per day for 4 consecutive days while their brain activity was recorded using electroencephalography. While our previous study has reported the brain activity changes from test recordings conducted before and after the sound exposure, we here analyzed the data recorded during the exposure. We investigated learning dynamics across daily exposure sessions using event-related potentials and multivariate pattern analysis and within sessions using a sliding average across trials. Both mismatch negativity and P3a—markers of change detection and preattentive attention shifts—exhibited learning-related changes in both the event-related potential analysis and multivariate pattern analysis. Changes in multivariate pattern analysis were evident after the first 2-h training session, while event-related potential–based effects emerged later. During the daily exposure sessions, the mismatch negativity amplitude gradually decreased over the first 3 d, whereas the P3a amplitude exhibited an opposite trend, showing a significant increase, and only on day 1. These findings demonstrate dynamic neural changes driven by passive exposure and pave the way for investigating learning processes across multiple levels of analysis, including event-related potential, single-trial dynamics, and machine learning–based methods.

## Introduction

In early life, the human brain becomes specialized in encoding native speech sounds, allowing for more efficient language processing (eg Kuhl 2004; Partanen et al. 2013). As a consequence of this development, perception of foreign speech is more difficult than that of native speech (eg Baills et al. 2019; Pelzl et al. 2019, 2021). However, with practice, adults can learn to discriminate foreign phonetic contrasts, which is also reflected in changes in brain activity (eg Golestani and Zatorre 2004; Zhang et al. 2009; Yi et al. 2021).

Numerous studies have applied change detection paradigms to study perception and acquisition of non-native speech contrasts (eg Kirmse et al. 2008; Ylinen et al. 2010; Saloranta et al. 2020; Yang et al. 2024). In these studies, event-related potentials (ERPs), namely the mismatch negativity (MMN) and P3a, are elicited in a passive oddball condition reflecting deviance detection and attentional shifts toward deviant sounds, respectively (Näätänen et al. 1978, 2007). In various training conditions, after a single training session within 1 d (eg Tremblay et al. 1998) or multiple sessions during several days (eg Ylinen et al. 2010; Chobert et al. 2014; Højlund et al. 2022), an increase in the amplitude of MMN/P3a and/or a shortening of their latency have been observed. Our previous study (Kurkela et al. 2019) demonstrated that mere passive exposure to foreign speech sounds can induce plastic changes in brain activity related to deviance detection. However, like most prior research, it focused on outcome-based learning measures, neglecting the neural dynamics occurring during the learning process. As a result, previous studies have been unable to quantify the rate of these changes or determine whether they follow a linear trend during the learning process. While studies on novel word learning have reported rapid enhancements in neural responses after approximately 30 min of passive exposure (Kimppa et al. 2015), comparable investigations into real-time brain activity during phonetic learning of foreign speech sounds remain lacking.

Here we report results from a study where adult native Finnish speakers were passively listening repetitions of vowel /a/ with changes in its Chinese lexical tone. Sounds were presented in a passive oddball condition for 2 h per day for 4 consecutive days resulting in 8 h of passive exposure for each participant. Learning results based on test measurements conducted before and after the passive exposure have been published earlier (Kurkela et al. 2019), showing shortened MMN and P3a latencies, as well as increased P3a amplitude following the exposure. Here we report results on brain activity changes during the passive exposure sessions: interday and within-day learning dynamics in the neural activity patterns.

We expect that traditional ERP analysis will show an increase in P3a amplitude from the first day to subsequent days, but not necessarily in MMN amplitude (Kurkela et al. 2019). Exposure-related changes may also be observable in the ERP latencies (Kurkela et al. 2019). In the absence of similar previous studies, it is not possible to hypothesize whether they occur immediately after the first day or only after subsequent days of exposure. In addition to traditional ERP analysis, we employed multivariate pattern analysis (MVPA) to examine changes in neural activity patterns over time. MVPA has been recently utilized in electroencephalography (EEG) studies (eg Cauchoix et al. 2014; Huang et al. 2021; Ody et al. 2023; He et al. 2024), and it uses information at trial-by-trial resolution and whole-brain activity, or a selection of electrodes, to separate neural activity patterns (here EEG amplitude) over time (Fahrenfort et al. 2018; Treder 2020; López et al. 2022), making it potentially more sensitive to neural activity changes than univariate ERP analysis (eg Cauchoix et al. 2014; He et al. 2024).

We also investigated changes in brain activity within each 2-h exposure session. Given that larger MMN and P3a amplitudes are associated with phonetic learning (eg Chobert et al. 2014), we expected these components—particularly P3a, which showed an increase in pre–post measurements (Kurkela et al. 2019)—to increase as passive exposure progressed.

## Materials and methods

### Participants

Eighteen volunteers (mean age = 21.7 years, SD = 1.7) were recruited from among the students at the University of Jyväskylä. Inclusion criteria were age between 18 and 30 years, right-handedness, normal hearing measured with an audiometer, and self-reported normal/corrected to normal vision. Exclusion criteria were neurological and psychiatric disorders, sleep problems, tonal language learning or more than a 2-week stay in a tonal language environment. Written informed consent was obtained from all the participants before their participation. The study protocol was approved by the Human Sciences Ethics Committee of the University of Jyväskylä, and the study was conducted in accordance with the Helsinki Declaration.

Data from 4 participants were excluded due to missing triggers in the EEG recording during exposure, leaving 14 participants (mean age = 21.6 years, SD = 2.5, 11 females, 3 males) for the analysis. In the final sample, 57.1% of the participants had musical training or regarded music as a hobby, and all the participants had studied English and Swedish as foreign language at school. In addition, 64.3% had studied another foreign (nontonal) language for more than 2 years.

### Stimuli and procedure

Lexical tones, a non-native phonetic feature for the participants, served as the target of exposure. The original sounds were recorded from a female native Chinese speaker and edited digitally using SoundForge and Praat software, so pitch tier transfer was conducted to isolate lexical tones while preserving other acoustic characteristics. Pitch tier transfer generated a rising tone and a falling tone, which were identical to each other, except for a pitch contour difference in fundamental frequency (F0). These two tones were taken as the endpoint stimuli to create a continuum of lexical tones with 10 interval steps. The standard sound was a falling tone, while deviant sounds were a slightly falling tone (small deviant) and a rising tone (large deviant) corresponding to the tone tokens 11, 7, and 3, respectively, on the tone continuum (see fig. 1B in Kurkela et al. 2019). Each speech sound had a duration of 200 ms. For more details on the stimuli, see Xi et al. (2010).

Participants were exposed to speech stimuli for 2 h per day over 4 consecutive days, accumulating a total of 8 h of exposure. During the exposure, a total of 11,000 stimuli were presented in the oddball paradigm, with 80% of standard sounds and 10% of each deviant sound, using E-Prime software (version 1.2., Psychology Software Tools Inc., Sharpsburg, United States). Consecutive deviant sounds were separated by at least two standard sounds. The interstimulus interval (offset to onset) varied randomly between 440 and 520 ms. The sounds were presented through a loudspeaker positioned approximately 50 cm above the participant’s head, at a sound pressure level of 50 dB. Participants were instructed to ignore the sounds and focus on watching a movie. No information about the sounds was provided at any point. To ensure sustained attention to the movie, a break was taken every 30 min, during which participants were asked questions about the movie’s plot. The second, third, and fourth exposure sessions, conducted on consecutive days, followed the same procedure as the first session.

During the speech sound exposure, EEG was recorded with NeurOne amplifier (Bittium Biosignals Ltd, Kuopio, Finland) using a 32-channel sensor cap (Electro-cap International Inc., Eaton, Ohio, extended 10-to-20-electrode system) where 21 electrodes were used: Fp1, Fp2, F3, F4, F7, F8, Fz, C3, C4, Cz, P3, P4, Pz, T3, T4, T5, T6, O1, O2, Oz, and A2. The left mastoid served as the reference electrode, and the ground electrode was placed on the forehead. Data were sampled at 500 Hz with a band-pass filter between 0.1 and 200 Hz. Impedances were kept below 5 kΩ.

### Electroencephalography preprocessing

Data preprocessing and analysis were performed using EEGlab (Delorme and Makeig 2004) and FieldTrip (Oostenveld et al. 2011) toolboxes, together with custom-written MATLAB (The MathWorks, Inc., version R2019a) codes (the scripts can be found at AsophiliaChance/Passive-exposure-learning-dynamic [github.com]. Raw data were downsampled to 250 Hz, low-pass filtered at 30 Hz, and notch filtered at 50 Hz. Muscle activity artifacts were rejected based on spectrum thresholding (10 dB) from 35 to 128 Hz. Artifact components were identified for the continuous data using independent component analysis decomposition and those identified as eye movement components were removed from the data. Data were segmented for −100 to 600 ms after stimulus onset. Baseline correction was calculated by subtracting the average of the prestimulus window of −100 to 0 ms from the entire segment. Only the responses to standard stimuli occurring just before deviant stimuli were included in the averages, ensuring an equal number of standard and deviant responses. This balance was especially crucial for the single-trial analysis. Segments with amplitudes exceeding ±200 μV for a deviant/standard and its corresponding standard/deviant stimulus were rejected. Data were re-referenced to linked mastoids and averaged for each subject and stimulus type. The differential response reflecting change detection was obtained by the responses to the deviant stimuli minus the responses to the standard stimuli.

### Interday analysis

#### Event-related potential analysis and statistics

Based on findings from our previous study (Kurkela et al. 2019), which analyzed pre- and posttest recordings, and a visual inspection of the current passive exposure data, we selected responses from frontal electrodes (F3, F4, and Fz) for the analysis, as these sites exhibited the strongest MMN and P3a responses. We used averaged responses over these electrode sites in the ERP analysis. To define time windows for MMN and P3a amplitude analyses, we first constructed a grand average differential waveform (deviant minus standard) by averaging data from the frontal electrodes across all 4 exposure days, both deviant types, and all participants. The MMN peak amplitude was defined as the most negative point in the grand-averaged waveform, and the P3a peak as the most positive. Time windows for analysis were defined as ±35 ms around each peak: 189 to 259 ms after stimulus onset for MMN and 281 to 351 ms after stimulus onset for P3a. For each participant, amplitude was calculated as the mean voltage within the corresponding time window, and latency was determined based on the timing of the peak within that window.

To examine changes in ERP activity relative to day 1, linear mixed-effects models (LMMs) were applied to each participant’s averaged ERPs using MATLAB R2019a (MathWorks, Natick, MA, United States) with the fitlme function and related tools from the Statistics Toolbox (for a previous ERP study using an LMM, see Hickey et al. 2025). LMMs were fitted separately for the amplitude and latency of the MMN and P3a components, for each of the two deviant types. The models included day (days 2, 3, and 4, with day 1 as the reference) as a fixed effect, and participant as a random effect with random intercepts and slopes for day to account for individual variability. Models were estimated using maximum likelihood to evaluate the significance of fixed effects. The Satterthwaite approximation was used to calculate degrees of freedom and *P* values, ensuring robust statistical inference.

#### Multivariate pattern analysis

MVPA was employed to compare brain activity between the first day and subsequent days. The MVPA-Light toolbox (Treder 2020), implemented in MATLAB, was used for this purpose. To facilitate computational efficiency, the differential EEG responses were downsampled to 50 Hz.

We employed a temporal decoding approach (ie decoding across time; Treder 2020) using MVPA to identify time points in the EEG signal that reflected learning effects (eg Fahrenfort et al. 2018; Treder 2020; López et al. 2022). A linear discriminant analysis (LDA) classifier was trained independently at each time point within the EEG epoch to discriminate neural activity between exposure days. At each time point, we constructed a feature vector from the amplitude values of individual (single-trial) EEG responses recorded at three frontal electrodes (F3, Fz, and F4). This approach ensures that classification is based on time-resolved data, rather than on averaged ERP waveforms or whole-epoch features.

We conducted three binary classification analyses (day 1 vs day 2, day 1 vs day 3, and day 1 vs day 4) across the time window from −100 to 600 ms relative to stimulus onset. Classification performance was evaluated using 5-fold cross-validation, repeated 100 times to obtain a stable estimate. Performance at each time point was quantified using the area under the curve (AUC) metric.

Mean AUCs were computed for the MMN (189 to 259 ms) and P3a (281 to 351 ms) time windows and tested separately using one-sample *t* tests (one-tailed, Bonferroni-corrected, 10,000 permutations) to determine whether the classification performance significantly exceeded the chance level (AUC = 0.50). Neural patterns between the compared days were interpreted as being significantly different if *P* values were smaller than 0.05.

### Within-day analysis

#### Sliding average analysis and related statistics

We developed an analytical approach using difference waves to compute sliding ERP amplitude averages across bins of 100 trials, progressing in one-trial steps. Given that the smallest individual dataset contained 508 trials after artifact rejection, we generated 400 overlapping ERP averages per participant and deviant type (eg trials 1 to 100, 2 to 101, 3 to 102, …, 401 to 500). Consistent with the analysis investigating the between-day changes, ERP responses were averaged across electrodes F3, Fz, and F4, and mean amplitudes were extracted within two predefined time windows: 189 to 259 ms for MMN and 281 to 351 ms for P3a.These amplitude values were used to track changes in MMN and P3a responses during each daily exposure period. To quantify these trends, we fitted linear regression models to participant-averaged data, estimating the slope of amplitude change separately for MMN and P3a across each day and deviant type. A positive slope indicates a shift toward more positive polarity, whereas a negative slope reflects a shift toward more negative polarity. One-sample *t* tests were used to determine whether the slopes differed significantly from 0 (*α* = 0.05). Model fit was evaluated using the coefficient of determination (*R*^2^), which measures the proportion of variance in the dependent variable explained by the model and reflects the strength of the observed trend. Here, a statistically significant slope (*P* < 0.05) combined with an *R*^2^ value greater than 0.5 was considered as a clear trend in the data.

## Results

### Event-related potential analysis

**Fig. 1:**
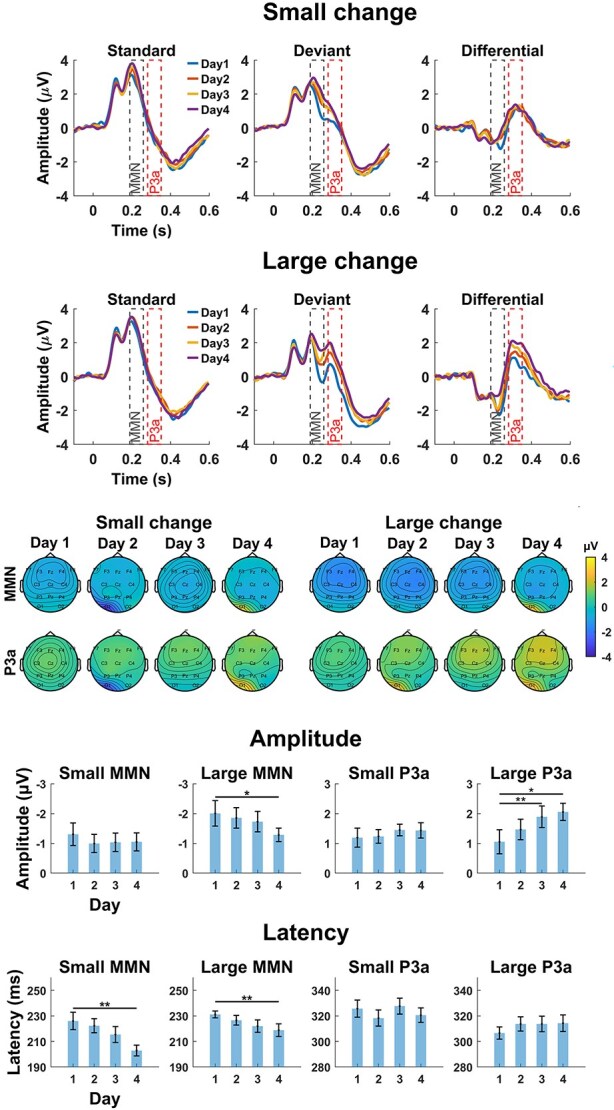
ERP waveforms, their topographies, and results of the amplitude and latency analyses of the exposure effect over the days. Top: grand-averaged ERPs on the 4 exposure days: Standard, deviant, and differential responses (deviant—standard) separately for small and large deviants. Waveforms represent mean values of the frontal electrode cluster (F3, F4, and Fz). The time windows applied for the MMN and P3a amplitude analysis (189 to 259 and 281 to 351 ms after stimulus onset, respectively) are marked to the figures. Please note that the differential responses were used in the analyses. The middle panel shows scalp topographies of the differential responses calculated as mean amplitudes across the respective time windows for MMN and P3a. The bottom panel shows the mean and standard error of the differential responses for both amplitude and latency. Asterisks indicate statistical significance based on linear mixed-effects models: ^*^P < 0.05, ^**^P < 0.01.

Statistical results for the amplitude and latency of MMN and P3a are summarized in Table 1, with corresponding waveforms, topographical maps, and bar charts presented in Fig. 1. For amplitude, the MMN response to large change was significantly reduced on day 4 compared to day 1. In contrast, the P3a amplitude in response to large deviants was significantly increased on days 3 and 4 compared to day 1. For small change, neither MMN nor P3a components showed significant amplitude changes across days. Regarding latency, MMN responses to both small and large deviants were significantly shorter on day 4 relative to days 1 and 2. No significant latency changes were found for P3a responses.

**Table 1:** Results of linear mixed-effects models assessing the impact of exposure on MMN and P3a amplitude and latency. Analyses were conducted separately for each deviant type (small and large), comparing brain activity on each subsequent day (days 2 to 4) to that on the baseline (day 1).

				**Estimate**	**SE**	***t***	***P***
**Amplitude**	**MMN**	Small	Intercept	−1.30	0.37	−3.57	0.003^**^
			Day 2	0.31	0.25	1.24	0.237
			Day 3	0.27	0.25	1.09	0.292
			Day 4	0.25	0.24	1.05	0.312
					
		Large	Intercept	−2.00	0.41	−4.88	<0.001^***^
			Day 2	0.15	0.22	0.67	0.514
			Day 3	0.28	0.23	1.22	0.244
			Day 4	0.72	0.30	2.37	0.032^*^
						
	**P3a**	Small	Intercept	1.19	0.31	3.90	0.002^**^
			Day 2	0.04	0.18	0.22	0.828
			Day 3	0.26	0.24	1.07	0.302
			Day 4	0.24	0.35	0.69	0.500
					
		Large	Intercept	1.05	0.39	2.72	0.017^*^
			Day 2	0.41	0.22	1.89	0.080
			Day 3	0.84	0.24	3.51	0.003^**^
			Day 4	1.00	0.38	2.60	0.021^*^
**Latency**	**MMN**						
		Small	Intercept	0.23	0.01	34.51	<0.001^***^
			Day 2	0.00	0.00	−0.99	0.337
			Day 3	−0.01	0.01	−1.83	0.088
			Day 4	−0.02	0.01	−3.82	0.002^**^
					
		Large	Intercept	0.23	0.00	89.15	<0.001^***^
			Day 2	0.00	0.00	−1.46	0.166
			Day 3	−0.01	0.00	−2.11	0.054
			Day 4	−0.01	0.00	−3.79	0.002^**^
						
	**P3a**	Small	Intercept	0.33	0.01	50.71	<0.001^***^
			Day 2	−0.01	0.01	−1.20	0.249
			Day 3	0.00	0.01	0.24	0.811
			Day 4	−0.01	0.01	−0.84	0.416
					
		Large	Intercept	0.31	0.00	67.17	<0.001^***^
			Day 2	0.01	0.01	1.30	0.216
			Day 3	0.01	0.01	1.08	0.297
			Day 4	0.01	0.01	1.15	0.269

### Multivariate pattern analysis

**Fig. 2:**
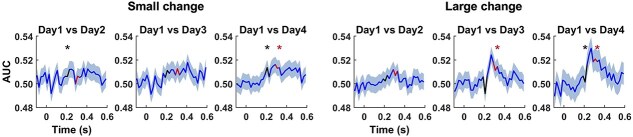
Statistical results of decoding (MVPA). Each curve shows the average AUC of differential response (deviant–standard), *n* = 14, with the shaded area representing the standard error. The color-coded segments indicate the MMN (the earlier latency) and P3a (the later latency) time windows, and the asterisks above them denote a significant above-chance-level decoding accuracy.

The results of the MVPA analysis are presented in Fig. 2. For the MMN component, responses to the small change differed significantly between day 1 and both day 2 and day 4, while responses to the large change showed a significant difference between day 1 and day 4. In contrast, the P3a response to the small change did not exhibit above-chance classification performance. However, for the large change, P3a responses demonstrated significantly above-chance classification between day 1 and both day 3 and day 4.

### Changes during each exposure session: sliding average analysis

**Fig. 3:**
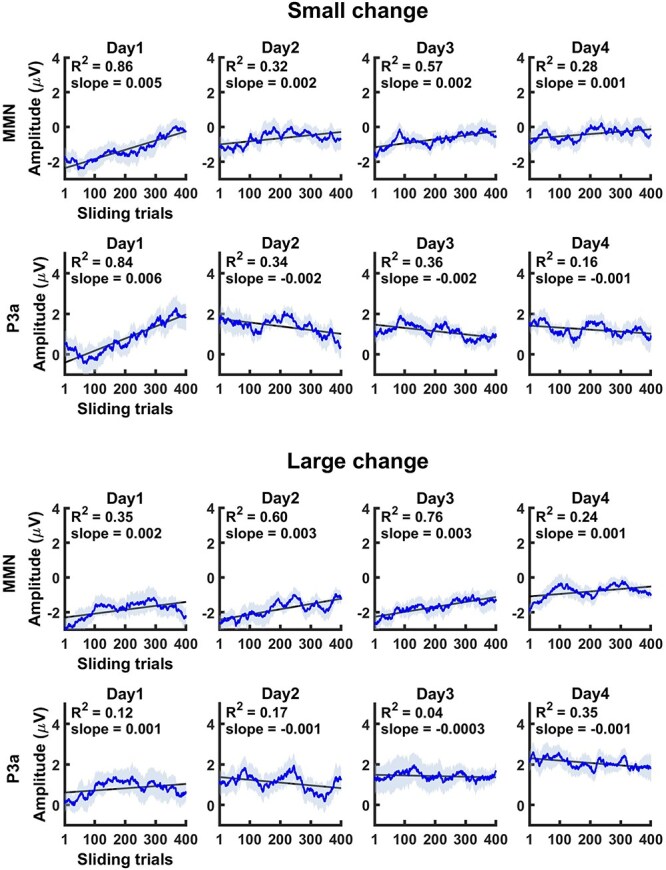
Results of the sliding average analysis. Each curve represents the mean amplitude of the sliding ERPs (differential response) across 14 participants. The shading of the curve indicates the standard error of the mean (SEM).

The results of the sliding average analysis are presented in Fig. 3. The slope of the response amplitude was statistically significant for both MMN and P3a components, as well as for both small and large stimulus changes, across all days—although some slope values were notably small. A clear trend, defined as a statistically significant slope combined with an *R*^2^ value greater than 0.5, was observed for MMN and P3a responses to small changes, and for MMN responses to large changes. Specifically, MMN amplitudes in response to small changes decreased on days 1 and 3, with *R*^2^ values of 0.86 and 0.57, respectively. In contrast, P3a amplitudes to small changes increased on day 1, with an *R*^2^ value of 0.84. For large changes, MMN amplitudes showed a decrease on days 2 and 3, with *R*^2^ values of 0.60 and 0.76, respectively. No clear trends in P3a amplitude were observed on any day in response to large deviants.

## Discussion

The ability to learn new phonetic features is essential for foreign language acquisition, yet the neural dynamics involved in this learning process remain underexplored. Additionally, existing studies have primarily focused on active learning scenarios, creating a gap in understanding how passive exposure contributes to phonetic learning. Addressing these gaps in the literature, our study investigated how a 4-d (2 h per day) passive exposure in adults impacts electrical brain responses associated with phonetic learning as indexed by the MMN and P3a components. ERPs revealed an increase in P3a amplitude and a concurrent decrease in MMN amplitude across the 4 d. Latency decreased for the MMN, but not for the P3a component. A machine learning–based method, MVPA, demonstrated that passive exposure to novel phonetic features can induce measurable changes in brain activity within just 2 h. Specifically, MVPA revealed that the brain activity patterns associated with the MMN response differed between the first and second exposure sessions, while changes in P3a occurred later. Analysis of response amplitudes during the daily 2-h passive exposure sessions revealed distinct patterns for MMN and P3a amplitude. Next, we will discuss these results in detail.

Using traditional ERP analysis based on participant-averaged trials from each daily exposure session, we found amplitude changes in response to large deviants: a reduction in MMN from day 1 to day 4, and an increase in P3a from day 1 to days 3 and 4. No significant amplitude effects were observed for small deviants. For latency, MMN showed decrease for both change types (day 1 vs day 4), but no P3a latency effects were observed. This pattern is mostly in line with our previous study, which involved partly the same subjects and utilized pre and post measurements with the same stimulus paradigm in a passive exposure context (Kurkela et al. 2019). In this previous study (*n* = 18 in the exposure group), we found that MMN and P3a latencies shortened, and P3a amplitude increased from pre to post measurement. However, no changes were observed in MMN amplitude or behavioral accuracy, and behavioral response times showed no significant change in the exposure group compared to the control group, which had no speech exposure but participated only in the ERP tests. Even though there were differences in recording and analysis methods between these two studies, some key findings remain consistent. Here we had only 14 participants’ data available for the analysis of exposure data, but longer stimulation time than in the pre and post measurements. Furthermore, exposure data were recorded with 21 electrodes, while 128 electrodes were used in the pre and post measurements. As a result, the reference electrodes were different. Despite these variations, both studies showed an increase in P3a amplitude, with the pre–post data showing no change in MMN amplitude, while the exposure data revealed a significant decrease in MMN amplitude. It is notable that even if the amplitude change for MMN was not significant in Kurkela et al. (2019), the direction of the MMN amplitude change was the same, decrease, as in the exposure data reported here. Some previous studies have also reported a reduced MMN amplitude following perceptual learning of lexical tones (Kaan et al. 2008; Lu et al. 2015). These studies have interpreted the reduction in the MMN amplitude as an indication that non-native speakers exhibit sensitivity to the fundamental frequency (F0) of tonal speech sounds prior to learning, which diminishes as they become more attuned to the phonetic features through learning. This interpretation could also apply to our findings.

The latency results were similar between the pre–post and exposure datasets for MMN latency. However, for P3a latency, we observed an exposure effect only in the pre–post dataset. This difference may be due to the differential responses used in this study. Peaks could not be reliably defined for each individual from the differential responses. In the pre–post data, where the P3a latency decrease was observable, we used only deviant responses to detect the peaks. In contrast, we used the differential responses in this study, which reduced the number of levels in the analyses. This approach was necessary due to the small sample size. In sum, our previous study (Kurkela et al. 2019) found ERP changes in MMN and P3a related to exposure, and the current study investigating the ERPs during the exposure revealed that the changes in them occur on the third and fourth days of exposure.

The MVPA based on single EEG epochs and time points revealed learning-related amplitude changes in both the MMN and P3a time windows. Temporal decoding analysis showed significant differences in MMN for small change between day 1 and day 2, and between day 1 and day 4. For the large change, differences were observed between day 1 and day 3, as well as between day 1 and day 4. No above-chance classification was found for P3a in response to the small change. However, for the large change, P3a showed significant above-chance classification between day 1 and days 3 and 4, as well as between day 2 and day 4. These findings suggest that the brain’s automatic detection of smaller changes in stimuli (captured by the MMN component) evolved more quickly over the exposure period, indicating rapid learning or adaptation. The MMN reflects an automatic, preattentive neural response to deviant or unexpected stimuli and is thought to be involved in the brain’s early detection of change (Näätänen et al. 1978, 2007). In contrast, the brain’s response to larger changes (captured by the P3a component) evolved more gradually. The P3a is typically associated with attention shifting (Näätänen et al. 1978, 2007), and it may require more time to show significant changes in response to larger deviations. It is possible that smaller changes in stimuli may not evoke as strong or as consistent neural responses—particularly in terms of P3a—compared to larger changes. The difference in how these components evolve over time may reflect distinct aspects of learning: MMN changes associated with early detection of subtle differences and P3a changes tied to shifting attention to more noticeable stimuli.

We also examined amplitude changes in MMN and P3a amplitude within each daily exposure session. Due to the difficulty of reliably identifying peak latencies for the differential responses at the single-trial level, only amplitude data were analyzed. Within a 2-h session, the MMN amplitude reduced on days 1 and 3 for small change, and on days 2 and 3 for large change, while P3a enhanced, but only on day 1 for small change. Notably, while the traditional ERP analyses showed an increase in the P3a amplitude to large changes from day 1 to days 3 and 4, no robust amplitude increase was observed during any of the exposure sessions themselves. Visual inspection of amplitude values (Fig. 3) suggests that large change consistently elicited higher P3a amplitudes at the beginning of each daily exposure session. Similarly, although the MMN amplitude to large change showed a decreasing trend from day 1 to day 4 in the ERP analysis, the sliding average data indicate a reduced MMN amplitude at the beginning of the exposure session on day 4. It is thus possible that memory traces were consolidated during nocturnal sleep, leading eventually to amplitude increases observed between the exposure sessions. Future studies should investigate the role of memory consolidation in perceptual learning, as most existing research has focused primarily on associative learning (for a review, see Diekelmann et al. 2009; for perceptual learning of speech sounds, see eg Fenn et al. 2003). Changes over the sessions were more clearly observed for small rather than large changes. This may be explained by the fact that large changes were easier to detect than small changes—on average, 97.0% of large changes and 88.4% of small changes were detected in the baseline recording of the active change detection task in Kurkela et al. (2019)—leaving more room for perceptual learning in response to small changes.

As this study represents the first attempt to explore ERP dynamics of learning foreign speech sounds over extended periods of passive exposure, future research should investigate the factors influencing the amplitude changes during prolonged recordings and whether distinct response patterns are associated with learning outcomes. This would require a larger sample size allowing correlational analyses. Here the limitation was that no behavioral responses were available due to the nature of exposure (passive listening), and because of the small sample size it was not possible to correlate the brain activity changes recorded during the exposure with the behavioral change detection investigated in pre and post tests in the context of the active oddball condition. In addition, in the previous study reporting the pre–post test results, no improvement in behavioral accuracy was found (Kurkela et al. 2019).

In summary, both ERP- and machine learning-based methods (MVPA) detected changes in brain activity during passive exposure to foreign speech sounds. MVPA revealed neural changes after just the first 2-h session, whereas changes in ERP components emerged across the subsequent days. Within-session effects were most pronounced in the ERPs on the first day of exposure. The findings suggest that passive exposure to foreign speech sounds can rapidly induce neural plasticity. However, future studies are needed to better understand the neural changes underlying both the machine learning and ERP findings, and how they may be linked to behavioral-level speech perception. Investigating the time course and both cortical and subcortical mechanisms of passive speech sound learning could help optimize exposure protocols for more efficient language acquisition.

## Data Availability

The raw data are not publicly available due to legal restrictions, as the participants did not provide consent for data sharing. The data that support the findings of this study are available upon reasonable request from Piia Astikainen (piia.astikainen@jyu.fi). All custom-written Matlab codes are publicly available at: AsophiliaChance/Passive-exposure-learningdynamic (github.com).
